# Women's Perceived Reasons for Their Excessive Postpartum Weight Retention: A Qualitative Interview Study

**DOI:** 10.1371/journal.pone.0167731

**Published:** 2016-12-09

**Authors:** Anne Christenson, Eva Johansson, Signy Reynisdottir, Jarl Torgerson, Erik Hemmingsson

**Affiliations:** 1 Obesity Center, Karolinska University Hospital, Department of Medicine, Karolinska Institutet, Stockholm, Sweden; 2 Department of Public Health Sciences (IHCAR), Karolinska Institutet, Stockholm, Sweden; 3 Department of molecular and clinical medicine, Institute of Medicine, Sahlgrenska Academy, University of Gothenburg, Göteborg, Sweden; 4 Department of Medicine, Karolinska Institutet, Stockholm, Sweden; University of Tennessee Health Science Center, UNITED STATES

## Abstract

**Introduction:**

Obesity in Sweden has doubled to 14% over the last 20 years. New strategies for treatment and prevention are needed. Excessive gestational weight gain has been found to contribute substantially to obesity, and there is a consistent association between postpartum weight retention and obesity later in life. We aimed to explore what factors women perceive as reasons for having substantial postpartum weight retention, to identify areas for new and improved interventions.

**Methods:**

Qualitative interview study (semi-structured) using an emergent design. Fifteen women, with a postpartum weight retention ≥ 10 kg, were interviewed by a trained cognitive therapist. Eight women had pre-pregnancy BMI below 30 kg/m^2^. Interviews were transcribed verbatim and data analysed using inductive manifest content analysis. Salient text passages were extracted, shortened, coded and clustered into categories.

**Results:**

Participants reported no knowledge of current gestational weight gain recommendations or of risks for adverse pregnancy outcomes with excessive weight gain or postpartum weight retention. Excessive eating emerged as a common strategy to provide relief of psychological, emotional and physical discomfort, such as depression and morning sickness. Women perceived medical staff as being unconcerned about weight, and postpartum weight loss support was scarce or absent. Some women reported eating more due to a belief that breastfeeding would automatically lead to weight loss.

**Conclusion:**

There is a need to raise awareness about risks with unhealthy gestational weight development and postpartum weight retention in women of childbearing age. The common strategy to cope with psychological, emotional or physical discomfort by eating is an important factor to target with intervention. The postpartum year is a neglected period where additional follow-up on weight and weight loss support is strongly indicated.

## Introduction

Over the last 20 years the prevalence of obesity in Swedish women has doubled to the current 14% [1]. With existing non-surgical interventions, including diet, physical exercise and weight-loss medications, only around 20% of obesity patients achieve a significant risk reducing weight loss (>10%) [2, 3]. New and improved ways of treating, and preferably preventing, obesity are needed.

For three out of four female patients in an obesity clinic the pregnancy period contributed to their obesity by retaining around 10 kg from each pregnancy [4]. One of the strongest predictors for postpartum weight retention is excessive gestational weight gain [5, 6]. Since 2009 the recommended upper limit for pregnancy weight gain, based on pre-pregnancy Body Mass Index (BMI), is 16 kg for normal weight, 11 kg for overweight and 9 kg for women with obesity [7]. Earlier guidelines from 1990 used different BMI cut-offs, categorizing more women as underweight or obese and fewer as normal-weight or overweight [8], and advised women with obesity to gain at least 6 kg with no upper limit mentioned [9]. Regardless of pre-pregnancy BMI more than 70% of women exceed the current recommendations [10, 11]. Even with intervention programs aimed to limit weight gain during pregnancy, about 40–60% of women still gain excessively [10, 12–14]. The limited success of weight management during pregnancy and the scarce existence of weight loss support postpartum is unfortunate since recent studies show that weight-retention of two BMI units or more increases the risks in subsequent pregnancies for gestational diabetes, hypertension, stillborn babies and early infant death [15, 16].

Research on predictors of excessive gestational weight gain and postpartum weight retention is limited and inconclusive. Predictors identified in available studies are: low self-esteem, anxiety early in pregnancy, depression and low socioeconomic status although the mechanisms remain unclear [17, 18]. We aimed to find out what women themselves perceive as reasons for their excessive weight gain and their inability to lose weight postpartum, so that more effective interventions can be developed.

## Methods

### Study Design

We chose to conduct a qualitative study with semi-structured interviews to explore a phenomenon where current knowledge is scarce and existing theories complex and diverse. Since the area of interest is only partially explored a qualitative study allows the participants and researchers to discover new and unknown areas without the limitations of predetermined quantitative questions.

Weight can be a sensitive subject. Overweight and obesity is a stigma and often involves shame that could limit women’s willingness to speak freely about their possible shortcomings or difficulties. To establish a good contact and comfortable atmosphere, interviews were carried out by the primary investigator, a female therapist with 20 years' experience of talking with clients about weight issues in a specialist obesity centre. Interviews took place where the women found it most convenient, which was either in their home, at a café, at the obesity clinic or via Skype. When developing the study design, we used the "Consolidated criteria for reporting qualitative research (COREQ): a 32-item checklist for interviews and focus groups" [19] to certify that a high quality report would be obtained.

### Study participants and recruitment

Fifteen Swedish speaking women, with experience of retaining ≥10 kg of their pregnancy-related weight gain one year post partum, were recruited between May 2015 and May 2016 via information leaflets at children’s care units, gyms, weight loss centres, nursery schools and via a monthly online newsletter distributed by a company that sells weight loss products. The period of research interest includes the whole period from conception to one year post partum (≈21 months). Women were included if no more than 8 years had passed after their first postpartum year to ensure their memories were as extensive and accurate as possible. Fourteen out of fifteen women were interviewed 0–4 years after the end of the postpartum year and one woman was interviewed 7 years after. Mean time elapsed, between the end of the postpartum year and interview, was 2.5 years, median: 2 years range 0–7 years. The women in the sample self-reported pre-pregnancy height and weight. Five women were of normal pre-pregnancy weight (BMI 18,5–24.9 kg/m^2^), three were overweight (BMI 25–29.9 kg/m^2^), and seven women were obese (BMI above 30 kg/m^2^), mean: 30.8 median: 28.7 range 23.0–47.4 kg/m^2^. Four women were pregnant before or during 2009. Two women had gestational weight gain within recommendations that were current at the time of their pregnancy. The sample selection was purposeful as we wanted as diverse a sample of study subjects as possible regarding age and ethnicity to cover and explore many different stories and experiences. Eligible women were interviewed in the order they applied. The variation in age and ethnicity among the women who applied was deemed sufficient and there were no drop-outs. Mean age at pregnancy was 31 years, range 19–40 years, and 5 of the 15 women (33%) were from other countries than Sweden, (22% in the general population) [20]. The interviewer had no former established contact with the participants apart from one woman who had been a former patient to the interviewer. Pilot interviews were conducted to test the interview guide, and since no revision was deemed to be necessary data from the pilot interviews were included in the study. All women received oral and written information from the interviewer about the research aim and procedures and provided written informed consent. The study was approved by the Research Ethics Vetting Board in Stockholm, Sweden nr. 2015-605-31/5.

### Data collection

To determine sample size, we used the data saturation approach, where the researchers determine when the data collected from the interviews becomes redundant and it is estimated that the inclusion of more study subjects would add little to understanding of the study phenomenon. Interviews were transcribed verbatim, read through and analysed continuously and women were recruited and interviewed until no new factors regarding the research aim and the topics below emerged. The interviews were semi-structured where the women were encouraged to elaborate on the following subject areas:

- Weight development during pregnancy
- Weight development the year after pregnancy
- Knowledge of weight recommendations during and after pregnancy
- Knowledge of risks for adverse events with unhealthy weight gain during pregnancy
- Expectations they might have had before pregnancy about how their weight development would be
- Beliefs and experiences around breastfeeding and weight
- Whether they have tried to limit their weight gain during or after pregnancy and if so in what way and with what result
- Psychological factors or circumstances around the pregnancy period
- Earlier weight issues, eating disorders or psychological difficulties
- What they think (if anything) could have helped them keep a healthier weight if they had the chance to go back in time
- What they think distinguishes them from women who return to pre-pregnancy weight
- Any other factors or circumstances they think may have influenced their weight development

Questions were open, e.g. "Tell me about your weight development during your pregnancy!" which let women bring up any factors they associated with that topic. The therapist probed with more questions when necessary to go deeper and clarify uncertainties. Women were encouraged to talk about any factors they thought had influenced their weight curve. This also allowed other relevant topics to surface than had been mentioned in the questions above. Throughout the interview the therapist made several summing up statements of what she had heard to make sure she had understood the participant correctly, e.g. “A: So then there were a lot of arguments and tension in your relationship? P: Mm (yes)”. The interviews comprised 14 face-to-face interviews and one Skype interview and lasted between 15 and 60 minutes. Interviews were audio-recorded and then transcribed after which the recordings were erased. Field notes were made by the interviewer to complement the transcribed interviews. The transcribed interviews were depersonalized and participants were provided a copy of the transcript to be able to correct or complement their statements. None of the women requested corrections or clarifications to their data.

Weight data were self-reported. Postpartum weight retention ≥10 kg was defined as the difference between pre-pregnancy weight and weight one year post partum. It is known that self-reported weight may be underestimated [21] but since we were interested in the weight difference we assumed that any underestimation women would make would be the same for both pre-pregnancy and the postpartum body weight.

### Data analysis

Manifest inductive content analysis was used in the process of identifying recurrent themes. Content analysis is a systematic way of analysing and describing a phenomenon by condensing large quantities of information into fewer content-related categories [22–24]. The process of analyzing data contained the following four steps: 1) *Immersing oneself in data*. The interview text data was transcribed verbatim and read through several times to get a thorough sense for the interview content. 2) *Selecting meaning units*. Sentences or text blocks (meaning units) that included information relevant to the research aim were highlighted. 3) *Condensing and labelling of data*. Highlighted meaning units from the original text were condensed (shortened) and then labelled with a code. 4) *Clustering and categorizing of codes* (Table 1). The analytic process, including the labelling and clustering of codes into categories (and choosing not to formulate themes) was done in close discussion among the researchers. The purpose of the categories was to present a condensed yet broad description of what areas influence women’s weight development.

**Table 1:** Example of the process of condensing and analysing text data.

Original text (meaning units) from interview	Condensed meaning units	Codes	Categories
"Some people are just born like that. They gain weight really easy so that (pause) not everybody does that /. . ./ My mum has always been fat so I've thought that's how it's supposed to be. That I can't be thin and that it (weight development) is out of my control"	Believes some people are born to gain weight easy. Since my mum is fat I thought I can't be thin	Gestational weight development is uncontrollable	Misconceptions
“. . .she said that the weight would just melt away when I breast feed (so I can allow myself to eat)"	Midwife said I would lose a lot of weight while breastfeeding	Breastfeeding always leads to substantial weight loss	
"I was always wondering. . .ok, the baby is in my belly but why does the rest of me get fat?"	Wondering why I get fat	Don't know why weight gain occurs	Lack of knowledge
”…and afterwards too, because you often get left adrift after you have given birth. There’s a lot of focus on the body and baby before you give birth but afterwards you become very alone”	After birth the focus is on the baby and you feel alone	Feeling left alone after delivery	Lack of support
"I also would have needed more support after delivery when food became my comfort while struggling with breastfeeding and depression!"	Wanted more support not to turn to eating for relief	Feeling left alone after delivery	

Translation from Swedish to English of the quotes used in the article were made by the author and checked by two English speaking peers.

## Results

The following five major categories indicate what reasons women perceived affected their incentive and/or inability to return to pre-pregnancy weight one year post partum (Table 2):

**Table 2:** Examples of codes clustered under subcategories and five major categories

Categories	Lack of knowledge	Misconceptions	Eating for relief		Lack of support	Barriers to physical activity
**Sub-categories**			**Psychological/emotional reasons**	**Physical reasons**		
**Codes**	Don't know why weight gain occurs	Gestational weight development is uncontrollable	Eating to feel calmer when stressed or anxious	Eating as a response to increased hunger	Feeling left alone after delivery	Pelvic joint pain
	Didn’t know how much weight gain was recommended	Breastfeeding always leads to substantial weight loss	Eating to feel less lonely and bored	Snacking decreased morning sickness	All focus is on the baby	Swollen feet
	Didn’t know what risks are involved with unhealthy weight development	Eating too much without concern or worries in the belief that it’s harmless to gain a lot of gestational weight	Eating for comfort when feeling depressed	Eating as a response to craving	Rejecting support because of shame for not being an able mother	Activity restrictions for medical reasons
				Eating to distract pelvic joint pain	Would have liked the info in writing, about what and how much to eat	
				Eating to feel more energetic when tired from lack of sleep	Would have liked physical activity groups initiated from health care	

### Lack of knowledge

None of the study participants expressed correct or detailed knowledge of either how much weight gain was recommended during pregnancy or what the risks were for adverse pregnancy outcomes or future obesity with excessive weight gain and postpartum weight retention. (A = Author P = Participant)

“A: Did you know anything about risks and health around pregnancy and weight?

P: No, not specifically for pregnancy but about being overweight. I know that it affects your heart and vessels and such.

A: You mean for you?

*P*: *Yes*, *exactly*. *But nothing about how it could affect the baby*. */*…*/ I don’t think it was mentioned that much and the midwife didn’t seem to put that much emphasis on it either”* (Woman 2)

“P:…well I know you’re not supposed to gain a lot cause it’s not good for your health.

A: Aha, ok. What were you thinking of in that regard?

P: Well, diseases, diabetes and such things…

A: What about those things?

P:…or amniotic fluid…isn’t it?

A: Ok…

*P*:*…but only in general terms…not something I can really say that I have knowledge of!”* (Woman 3)

Women reported that medical staff either did not bring up weight-related risks or that their providers toned down the risks with excessive weight gain and postpartum weight retention. At first visit most women were weighed while some only self-reported their weight and during pregnancy weighing was reported as scarce or sometimes absent and women felt midwifes put no particular emphasis on weight development.

“A: Did anyone ever talk to you about the risks of gaining excessive weight?

*P*: *No*, *actually not!”* (Woman 14)

“A: Did you have any…like…thoughts or ideas or knowledge about risks and weight gain around the pregnancy?

*P*: *No*…*and I was never weighed at the maternity clinic*. *The midwife asked about my weight at the admission visit so I told her my weight and then she asked one more time but otherwise nothing*. *It didn’t seem like anything she was worried about or put too much emphasis on*. *Therefore I didn’t think anything of it either”* (Woman 5)

Women claimed they were unaware of weight recommendations and what they risked by exceeding them and hence felt no worries or paid any attention to their weight or eating habits. None of the interviewed mothers had understood that their weight gain could have been dangerous to their child. Some were aware of the risk of getting diabetes but not of how diabetes ultimately can harm the pregnancy and the baby. This meant that women could indulge in eating without thinking too much about it and procrastinate about dealing with their weight.

*“During my first pregnancy I just ate what I wanted when I wanted it*. */*…*/ I thought I’ll deal with it (the weight) when the kid’s out”* (Woman 13)

*“When you’re pregnant you’re fat anyway so then it doesn’t matter if you eat (too much) because no one can notice /*…*/ so you can kind of eat away and it doesn't show anyway"* (Woman 2)

### Misconceptions

The misconception that gestational weight change is mostly predestined by genetic factors, and therefore less controllable, negatively affected the women's motivation to actively try to control their weight.

"My mum has always been fat so I've thought that's how it's supposed to be. That I can't be thin and.....that it (weight development) is out of my control." (Woman nr 2)

“Both my sisters gained over 25 kg so I just assumed that I would do that too and that it’s normal for people in my family” (Woman nr 14)

The belief that breastfeeding automatically leads to substantial weight loss post partum was strongly present in the stories of several women. Some women consciously let go of previous food restraints and ate whatever they wanted trusting that breastfeeding would automatically lead to substantial weight loss post partum. Others believed that one should eat more when breastfeeding. This reliance on breastfeeding for automatic weight loss was perceived as reinforced by friends, media and medical staff and gave women a false promise of easy postpartum weight loss that lowered their motivation to limit their weight gain during pregnancy or eat healthy post partum.

*"(woman quoting what she was told by her midwife) Don't worry! It will all melt away when you breast feed! "* (Woman nr 7)

### Eating for relief

Eating as a strategy to relieve psychological, emotional and physical discomfort such as: anxiety, depression, stress, boredom, restlessness, tiredness, pain, morning sickness, hunger or cravings, pervaded many stories and thereby emerged as a mediator for both psychological and physical factors on the pathway to excessive weight gain and/or weight retention.

“A: You mentioned stress. What are your thoughts about that?

P: Well the whole situation when we came back from abroad was…. Then he was very sensitive and that affected us all.

A: How did it affect you?

P: Well, it was the communication. He was very sad and got irritated and annoyed and I was constantly trying to… now I have a temper too so we had a lot of fights/…. /

A: So then there were a lot of arguments and tension in your relationship?

P: Mm (yes) /…/.

A: How did the stress affect you?

P: Well, I eat.

A: You eat?

*P*: *Yes*, *when I’m stressed I want to gorge*. *I walk around and I snack /…/I open the fridge*, *I open the cupboard and check what’s in it*. *Candy*, *ok*, *I take a bite and walk around again"* (Woman nr 3)

Some of the discomforts that lead to eating were directly connected to the pregnancy or motherhood, for example morning sickness, pelvic joint pain, increased hunger and tiredness due to taking care of the baby at night.

*“I ate very unhealthy because I was suffering from morning sickness for a long time in the beginning of the pregnancy so I never had any real regularity in my eating*. *The irregular eating made me feel more nauseous and then I tried eating to relief the nausea*… *I ate sweets because I felt less nauseous”* (Woman nr 5)

*“I ate a huge amount of carbohydrates*, *food that gave me energy*, *sugar of course*, *to be able to cope (stay awake)*. *I felt that when I was tired and slow I had to eat something*, *not because I was hungry but to get energy*. *I was aiming to be there for him (the baby) 24h/day”* (Woman nr 6)

Women expressed they were often painfully aware of their unhealthy behaviours but felt compelled to eat due to a lack of healthier coping strategies. Some of them claimed that, looking back, they think they must have had an undiagnosed post-natal depression that they self-medicated by eating sweets and chocolate to make it through the days.

"When I feel sad I just want to (showing with her hands how she's shoveling food into her mouth). Or if I'm eating I don't notice that I'm eating or if I'm full or…well, then I just eat. I just sit and eat.

A: Aha, so it's both that you eat because you feel sad and also that you don't really pay attention to what you eat when you're feeling down?

*P*: *Exactly (nodding)!"* (Woman 1)

*“*…*because for about a year I felt*…*it was just like darkness every day and everything was just really hard work /*…*/then I was very bored so then I just sat and ate*. *Food became sort of like a friend /*…*/ I feel good when I eat*. *It's a way to escape reality*. */*…*/ I feel better when I eat chocolate and sweet stuff because then I feel less depressed"*. (Woman nr 2)

*“yes*, *it’s sort of like*…*when the children have fallen asleep in the evening and you have that tiny gap in time before you need to go to bed yourself*. . . .*then my way of relaxing is to sit down in the sofa and eat something*. . . .*preferably candy or ice cream or sweets*” (Woman nr 5)

*"When I’m sad or bored it feels like there’s nothing else to do but to eat and you don't feel like going out so you isolate yourself and think that I’m fat anyway so I might as well (eat)”* (Woman 15)

### Lack of support

Several women expressed a feeling of being left on their own as soon as the baby was born. After delivery, the focus was exclusively on the baby and support for weight loss or psychological issues from medical care post partum was usually absent. Only one of the women received postpartum follow-up on weight and that was because she was obese at the beginning of pregnancy and thereby already included in an intervention program. Several women expressed a wish for more support from friends or their spouse and more proactive interventions from medical staff, for example a phone call or a follow-up visit including weighing and possible exercise groups organized by maternity care units.

”*…and afterwards too*, *because you often get left adrift after you have given birth*. *There’s a lot of focus on the body and baby before you give birth but afterwards you become very alone*” (Woman nr 2)

"I told my midwife that I was worried about my weight but she said -That's no problem! -It (the weight) will go away! I wish someone would have paid attention and pointed out to me and said:-It is time you pay attention to what you eat! I also would have needed more support after delivery when food became my comfort while struggling with breastfeeding and depression!"

(Woman nr 8)

One mother was offered counselling after delivering a child with a birth defect but wasn't able to take the initiative to make use of the offer. Another mother hid her feelings because of shame related to her own expectations of being a strong and able mother.

“I think I was depressed but I never showed it. I never sought help. There was a counsellor at the hospital and she gave me a card but it was just so silly to be told to “call if you need to”. They should follow up.

A: So you wish the initiative shouldn’t have to be yours but theirs?

*P*: *Yes*, *a call or visit or just turn up*, *and (they should) change the system*. *I mean house calls to check on how I am*. */…/ I wasn’t able to seek help and no one knocked on my door either*. */…/ I think it was a depression*. *If I would have sought help it may have taken shorter time (to get over the depression)*. (Woman nr 6)

*“When people asked how I was*, *I just answered that I was fine*. *It’s really hard to admit (that you feel depressed)*, *not even my partner noticed”* (Woman nr 2)

### Barriers to physical activity

Pelvic joint pain, feet so swollen that no shoes fitted, restrictions to stay in bed due to the risk of miscarriage and feeling too heavy in late pregnancy were barriers to physical activity during pregnancy. After delivery factors such as: tiredness due to lack of sleep or depression, cold weather, forgetting about own needs when focusing on the baby and remaining pelvic joint pain were barriers to physical activity. Women did not express having made any attempts to adjust their calorie intake to their lowered physical activity level.

*“My feet got so swollen…a lot of water so I walked around in sandals in February because I couldn’t fit in any other shoes.”* (Woman nr 2)

*"My pelvic joints caused a lot of pain and hindered physical activity"* (Woman nr 9)

*“The baby was born during the winter so I stayed inside a lot and was physically inactive*. *It was a legitimate reason not to go out in the cold with a small baby*. *I was in my “baby bubble”*. (Woman nr 7)

*“I had a cesarean with complications and had to go through follow-up surgery which lead to physical activity restrictions*.*”* (Woman nr 7)

*"When I feel depressed I don't feel like doing anything*. *I don't feel like going outside or to do anything active"* (Woman nr 2)

## Discussion

### Main findings

This study provides an overview of what women perceive as reasons for their excessive gestational weight gain and their inability to lose weight post partum.

Lack of knowledge, misconceptions, the use of eating for relief, lack of support and barriers to physical activity indicates areas where interventions could improve weight-related outcomes. These findings are supported by an earlier study with similar findings where barriers or enablers to a healthy lifestyle during pregnancy were found in the following areas: psychological, emotional, cognitive, interpersonal, and environmental [25].

### Lack of knowledge

Women are more motivated to perform necessary adjustments to their eating behaviours if they are aware of what they are risking in terms of adverse pregnancy outcomes and future weight problems of their own [26]. The participants of this study were unaware of pregnancy-related risks with unhealthy weight gain, and thereby lacked incentive to look at or adjust unhealthy behaviours.

One possible explanation to why these women lacked knowledge is that a confusion of older and newer advice could have been present since some of the women were pregnant before or during weight recommendations were updated in 2009 (7). However, in our study neither older nor current weight advice seem to have been communicated.

Secondly, there may have been communication around weight issues that were just not perceived or remembered by the women but their perceived lack of communication is consistent with earlier findings where women report little focus on their weight from medical staff during pregnancy [27–29].

Thirdly, we cannot say whether medical staff have a lack of knowledge about weight recommendations or if the seeming lack of weight concern is due to an inclination to avoid sensitive subjects. Studies show that midwives may be reluctant to address sensitive subjects such as weight and feel they lack special skills regarding nutrition advice and communication to work with weight-related issues [27, 30–32].

Finally, focus on obesity and pregnancy has increased during the last years [33] but women who start their pregnancy as non-obese (as many of the women in this study) have been of lesser concern [27] which could explain why weight concern from midwives seemed low even when women expressed worries about their excessive or rapid weight gain in the current study.

### The bridge between knowledge and behaviour

Health behaviour change is affected by a complex combination of ability, motivation and opportunity [34, 35] and therefore risk information alone is unlikely to induce behaviour change. The Health belief model, Social cognitive theory as well as the Capability-opportunity-motivation-behaviour framework all have in common that they describe that lifestyle change is influenced by perceived self-efficacy [36]. The Transtheoretical model also acknowledge the importance of self-efficacy and suggests that any behaviour modification is reached by moving through a series of stages [37]. Women enter pregnancy with various resources and may be in different stages of a process towards a healthier life style. It is therefore important that information is supplemented with individualized support. Providing a pregnant woman who is rapidly gaining excessive weight due to emotional eating with information about weight-related risks may otherwise do more harm than good, unless adequate support is provided. This implies that it may be beneficial to both increase and improve communication between midwives and pregnant women about weight recommendations and risks with excessive weight gain.

### Misconceptions

Despite a lack of scientific support women believed and were also told that breastfeeding would induce substantial weight loss. Findings about the influence of breastfeeding on weight loss are inconsistent [38]. Most studies show that breastfeeding has a positive, but small effect on post-partum weight loss, with a modest difference between non-breast feeding and breastfeeding of around 0.4–2.0 kg [39, 40]. Yet the belief in a much exaggerated effect on weight loss from breastfeeding was persistent and used by the women as an argument to procrastinate with lifestyle changes, and also by medical staff with the intention to calm down any weight worries the pregnant women may have. Providing appropriate information about the impact of breastfeeding on weight loss is a balancing act, knowing that being worried and anxious during pregnancy is associated with higher postpartum weight retention [17]. Tackling misconceptions may also be a challenge considering a recent study showed that behaviours during pregnancy were mainly based on knowledge and ideas accumulated before pregnancy, whereas health advice from medical staff exerted lesser influence [41].

### Eating for relief

The association between emotional eating and obesity is consistent with earlier findings [42, 43]. The pregnancy period with hormonal fluctuations, relationship issues and entering a new role as a mother can be emotionally challenging and stressful [44] which in turn may induce emotional eating [45]. Around 10–15% of women experience postpartum depression [46], which may contribute to weight retention, for example due to eating for relief. In this study the strategy of eating for relief emerged as a mediator not only for emotional or psychological factors but also to relief physical discomfort, hence our choice to call the phenomenon "eating for relief" rather than the commonly used "emotional eating". Increased hunger, cravings, morning sickness, pelvic joint pain and tiredness from sleepless nights post partum are common phenomena around pregnancy and without adequate knowledge, clear advice and appropriate support many turn to snacking for quick relief. Most women expressed knowledge about what constitutes a healthy diet and eating pattern but at the same time they reported lacking the ability to resist unhealthy eating when faced with emotional or physical distress. Excessive eating often functions as a way to relax, as distraction or as comfort [47]. This reveals an area where interventions may improve by offering therapeutic support to help women explore the underlying reasons and thereby better cope with emotional discomfort.

### Lack of weight loss support in the postpartum period

For some women their weight problems did not surface until after delivery and by then follow-up of the mother's health and weight was absent unless they had been obese to start with. Previous studies confirm that despite a normal BMI at the beginning of pregnancy and gestational weight gain within recommendations some women still experienced excessive postpartum weight retention [48]. Other studies show that successful weight limiting effects, from interventions during pregnancy, do not remain one year postpartum [49]. How we succeed in helping women back to pre-pregnancy weight (or lower if their BMI is higher than 25 kg/m^2^) affect the risk profile of any subsequent pregnancies as well as the risk for the mother to become obese. The lack of follow-up of the mother's weight is therefore unfortunate considering that many women have a second child. The postpartum period, which may very well be an inter-pregnancy interval, is an important but neglected window of opportunity for interventions [15, 50].

### Strength and limitations of the study

In terms of limitations, the time elapsed between the end of the postpartum year and the interview may have affected women's memories of circumstances and feelings around pregnancy. However, pregnancy is a very intense experience, and when it comes to reporting BMI and metabolic status it has been shown that women recall and report data with fair to excellent accuracy compared with objective data [51]. Fourteen out of fifteen participants were interviewed only 0–4 years after the end of the postpartum year and the information from the only woman with longer time elapsed did not differ from the other women's stories in terms of richness in details. Also we were aiming to explore their perceptions rather than objective events.

Psychiatric comorbidity with a variety of diagnoses is a known contributor to weight gain. Symptoms of psychiatric disorders or postpartum depression may emerge in the postpartum period and could be seen as a confounder [52]. None of the participants reported having been diagnosed with postpartum depression or psychosis but it cannot be excluded that some of the described difficulties could have been symptoms of an undiagnosed mild depression. It was beyond the scope of the current study to diagnose and consider postpartum depression as a separate factor. Instead we chose to look beyond any diagnoses to find underlying factors that are directly connected to eating or physical behaviours and thereby find concrete targets for interventions.

The author's interview skills from her many years of working with therapeutic sessions in an obesity clinic add strength to the study. Being a therapist may make the interviewer more susceptible to pick up topics of psychological nature. In this case the interviewer also has a background as a trained physiotherapist and had been working with obesity for over ten years before she became a cognitive therapist thus making it more plausible that both physical, psychological and other medical aspects were addressed both during interviewing and coding. We believe these skills facilitated for participants to speak openly about shortcomings and sensitive subjects while feeling comfortable and non-judged, allowing more data to be retrieved from the interviews.

Although the interview settings varied (home, clinic, café, Skype) the interview quality did not seem to differ. All interview modes contained privacy (no one could overhear the conversation) as well as the ability to see and hear each other well and thereby being able to pick up subtle changes in emotions or body language. The second author E J, who has actively participated in the study through the whole process, has extensive experience in qualitative research. In the process of writing the article the "Consolidated criteria for reporting qualitative research (COREQ): a 32-item checklist for interviews and focus groups" [19] was used to certify that answers to all items would be found in the report. Only item nr.28 in the COREQ checklist (“Did participants provide feedback on the findings?”) was not fulfilled since we decided to let participants only give feedback on the transcribed interviews.

## Conclusions

We need to raise the overall awareness in women of childbearing age about risks with unhealthy gestational weight development and postpartum weight retention. The common strategy to cope with psychological, emotional or physical discomfort by eating is an important factor to target with intervention. The postpartum year is a neglected period where additional follow-up on weight and weight loss support is strongly indicated.
